# Variation of Metagenome From Feedstock to Digestate in Full-Scale Biogas Plants

**DOI:** 10.3389/fmicb.2021.660225

**Published:** 2021-05-28

**Authors:** Fan Jiang, Sen Wang, Yan Zhang, Shichun Ma, Yan Huang, Hui Fan, Qiang Li, Hengchao Wang, Anqi Wang, Hangwei Liu, Lei Cheng, Yu Deng, Wei Fan

**Affiliations:** ^1^Guangdong Laboratory for Lingnan Modern Agriculture (Shenzhen Branch), Genome Analysis Laboratory of the Ministry of Agriculture and Rural Affairs, Agricultural Genomics Institute at Shenzhen, Chinese Academy of Agricultural Sciences, Shenzhen, China; ^2^Biogas Institute of Ministry of Agricultural and Rural Affairs, Chengdu, China; ^3^Laboratory of Development and Application of Rural Renewable Energy, Ministry of Agricultural and Rural Affairs, Chengdu, China

**Keywords:** metagenome, anaerobic digestion, feedstock, full-scale biogas plants, animal manure

## Abstract

Anaerobic digestion (AD) has been widely used to resolve the problem of organic wastes worldwide. Previous studies showed that the types of feedstock have a great influence on the AD microbiome, and a huge number of AD populations are migrated from upstream feedstocks. However, the changes of microbial compositions from feedstock to AD digestate are still less understood. We collected feedstock samples from 56 full-scale biogas plants, generated 1,716 Gb feedstock metagenomic data in total, and constructed the first comprehensive microbial gene catalog of feedstock containing 25.2 million genes. Our result indicated that the predominant phyla in feedstock are *Firmicutes*, *Bacteroidetes*, and *Proteobacteria*, which is similar to that in AD digestate, and the microbial diversity of feedstock samples is higher than that of AD digestate samples. In addition, the relative abundance of most genes involved in methanogenesis increase from feedstock to AD digestate. Besides, the amount of antibiotic resistance genes (ARGs) and pathogenic bacteria in AD are effectively reduced compared to feedstocks. This study provides a comprehensive microbial gene catalog of feedstock, and deepens the understanding of variation of microbial communities from feedstock to AD digestate of full-scale AD. The results also suggest the potential of AD to reduce the level of ARGs and pathogens in animal manure.

## Introduction

The vast amount of global organic waste due to population increase, urbanization expansion, and agriculture intensification has become a huge environmental burden (Tyagi and Lo, 2013). Recently, anaerobic digestion (AD) has been widely used to resolve the problem of organic wastes like animal manure, crop residues, and wastewater sludge and can produce biogas as renewable energy (Luo et al., 2016; Stolze et al., 2016). AD includes four sequential metabolic steps: hydrolysis, acidogenesis, acetogenesis, and methanogenesis (Angenent et al., 2004; Hassa et al., 2018), which are performed by highly complex consortium of microorganisms. In our previous metagenomic study on digestate of full-scale biogas plants (BGPs) fed with diverse feedstocks, we constructed a microbial gene catalog of AD (22,840,185 genes), and showed that the type of feedstock (cattle, chicken, and pig manure) has a great influence on carbohydrate hydrolysis and methanogenesis (Ma et al., 2021).

Feedstock for biogas plants contains a huge number of organic wastes, such as crop residues, animal manure, wastewater sludge, and food waste, and they were temporary stored in a tank before pumped into anaerobic digesters. During storage period, many spontaneous fermentation reactions were performed by microbes. Previous studies had indicated that a huge number of AD populations were migrated from upstream feedstocks in BGPs (Mei et al., 2017). However, the changes of microbial compositions from feedstock to AD digestate were less understood. Besides, since animal manure contains high level of antibiotic resistance genes (ARGs) and human pathogens, the manure directly applied as fertilizer will increase the dissemination of ARGs and pathogens to natural environment, which poses high risk to human health (Youngquist et al., 2016; Wu et al., 2018). Existing studies have proposed AD as a promising method to reduce some ARGs and pathogens in manure. However, whether AD can reduce all types of ARGs and pathogens remain to be verified.

In this study, we performed metagenome sequencing for corresponding feedstock samples from the 56 full-scale BGPs that were used in our previous metagenome study for AD digestate (Ma et al., 2021). These BGPs were operated at different temperatures, fed with diverse feedstocks, and distributed widely in geographical regions. We constructed a microbial gene catalog of feedstock, and compared metagenome of AD digestate and feedstock, to explore the variation of microbial communities from feedstock to digestate, as well as the changes of antibiotic resistance genes and pathogens during AD process.

## Materials and Methods

### Sample Collection

Feedstock samples were collected from 56 different full-scale biogas plants (BGPs) across 15 provinces of China, with one sample from each BGP. The characteristics of the 56 full-scale biogas plants were already described in our previous study (Ma et al., 2021). Feedstock samples were collected before pumped into anaerobic digesters, and 300 ml of feedstock were sampled from each BGPs and stored in 6 sterile, gastight tubes (50 ml). The samples were frozen immediately in a cooler with dry ice, and then transported to the laboratory. Frozen samples were stored at −80°C before DNA extraction.

### DNA Extraction, Library Preparation, and Sequencing

Genomic DNA of feedstock samples was extracted using the same protocol as for AD digestate samples (Ma et al., 2021), and all samples were processed together during the DNA extraction. Frozen feedstock samples were taken out from −80°C refrigerator and thawed at room temperature. Genomic DNA was extracted using PowerSoil DNA Isolation Kit (cat. no. 128880-100; MoBio Laboratories Inc., United States). The entire process was carried out according to the manufacturer’s instructions, with minor modification of adding an additional four freeze-thaw cycles (alternating between 65°C and liquid nitrogen for 5 min) before using the kit. The integrity of extracted DNA was checked on 0.7% (w/v) agarose gel with GelRed nucleic acid gel stain (cat. no. 41003; Biotium, United States). The quality and quantity of the extracted DNA were assessed using NanoDrop (Thermo Fisher Scientific, United States) and Qubit 2.0 Fluorometer (Thermo Fisher Scientific, United States). After DNA quality checks, the high-quality DNA (band length > 15 kb, A260/280 1.8–2.0, dsDNA concentration >20 ng/μL) of each sample were used for library construction. Sequencing libraries with 350 bp insert size were prepared using Illumina TruSeq DNA PCR-Free Library Preparation Kit (ref. 15037059; Illumina, United States) following the manufacturer’s instructions. Sequencing was performed on an Illumina Hiseq X10 platform (Illumina, United States) by Cloud Health Genomics Ltd (Shanghai, China) applying 150 bp paired-end strategy.

### Metagenome Assembly and Construction of the Gene Catalog

The metagenome assembly and construction of gene catalog for feedstock samples were performed using the same pipeline for our previous study (Ma et al., 2021). In brief, (1) raw reads were filtered by trimming the adapter contamination and low-quality bases using clean_adapter and clean_lowqual with default parameters, resulting in the clean and high-quality reads with an average error rate <0.001; (2) *de novo* assembly of the clean reads was carried out using Megahit (v1.1.3) (Li et al., 2016) under paired-end mode, with the preset “meta-sensitive” and the minimum contig length was set to 1,000 bp; (3) the assembled contigs were subjected to a gene prediction using the Prodigal software (v2.6.3) (Hyatt et al., 2012) with parameter “-p meta,” and filter out the genes with codon sequence length less than 100 bp according to a previous study (Qin et al., 2010); (4) the pooled genes from all 56 feedstock samples were then clustered to get the final non-redundant gene catalog using CD-HIT (v4.6.6) (Fu et al., 2012), with parameter “-c 0.95 –n 10 –G 0 –aS 0.9,” adopts the criteria of identity ≥95% and alignment coverage ≥90% of the shorter genes.

### Taxonomic and Functional Annotation of the Gene Catalog

Taxonomic and functional annotations of genes in the gene catalog of feedstock were performed using a previously established method (Ma et al., 2021). For taxonomic annotation, CARMA3 (Gerlach and Stoye, 2011) was used on the basis of DIAMOND (v0.8.28.90) (Buchfink et al., 2015) alignment against the NCBI-nr database. Functional annotations were made by DIAMOND searches against the KEGG database (release 79) (Kanehisa et al., 2004) by taking the best hit with an *e*-value < 1e-5. For the antibiotic resistance genes (ARGs) annotation, genes were predicted using Resistance Gene Identifier (v4.2.2)^1^ against the Comprehensive Antibiotic Resistance Database (CARD, v3.0.0) (Jia et al., 2017). The species with their scientific names included in Virulence Factor DataBase (VFDB 2019) (Liu et al., 2018), Global Pathogenic Bacteria Database (GlobalRPH), or CARD v3.0.0 (Jia et al., 2017) were identified as human pathogenic bacteria.

The relative gene abundance was calculated according to a previous study (Huang et al., 2018; Ma et al., 2021). To calculate the relative gene abundance of each sample, the clean reads of each sample were mapped separately onto the gene catalog by BWA-MEM, and the reads with alignment length ≥50 bp and identity >95% were defined as qualified reads. For each sample, total number of reads mapped to all genes (TA) equal to the count of qualified reads, total number of reads mapped to one gene (TO) equal to the count of qualified reads mapped to the gene. Considering the great differences of assembled gene length (GL) and mapped reads number (TA), the value of GL and TA were centralized by dividing 1,000 and 10,000,000 times respectively, since the average GL was about 1,000 bp and the level of TA was 10 million. At last, the normalized gene abundance (NGA) for each sample was calculated as followings: NGA = TO/(GL/1,000)/(TA/10,000,000). The relative abundance of different taxonomic ranks (superkingdom, phylum, class, order, family, genus, and species), KEGG orthologous groups (KOs), and ARGs were calculated by summing the relative abundance of the respective genes belonging to each category.

### Analysis of Metagenome-Assembled Genomes

Construction of metagenome-assembled genomes (MAGs) for feedstock samples was performed using the same protocol as for digestate samples (Ma et al., 2021). In brief, the reads of each sample were mapped back to the assembled contigs using BBmap v38.50^2^, and then the genomes were independently recovered from each sample by using the software of MetaBAT2 v2.12.1 (Kang et al., 2019), setting minimum scaffold length of 2,000 bp. Completeness (Cp) and contamination (Ct) of the MAGs were determined using CheckM v1.0.7 (Parks et al., 2015). De-replication of MAGs was performed according to their genome-wide average nucleotide identity (ANI) among different MAGs, and MAGs with ANI value >95% and genome coverage >50% were considered to belong to the same species, then the one with the highest CC3 value (CC3 = Cp – Ct ^∗^ 3) was selected as the representative. Finally, 2,101 MAGs were constructed for feedstock samples. Taxonomic annotation of these MAGs was performed with GTDB-Tk v1.3.0 (Chaumeil et al., 2019).

### Statistical Analysis

The microbial communities of feedstock were analyzed using the same protocol as published previously for analyzing the AD digestate from the same 56 BGPs (Ma et al., 2021). The relative gene abundance data of AD digestate for these 56 BGPs were cited from our previous study (Ma et al., 2021), and the data of all feedstock and AD digestate were then compared together. For microbial diversity analysis, the Shannon index was used. Box plots show medians ± interquartile ranges (IQR) and 1.5 IQR ranges (whiskers), with outliers denoted by open black circles. The overall differences in the bacterial community structures were evaluated by principal coordinate analysis (PCoA) based on Bray-Curtis dissimilarity values and performed by the R package PHYLOSEQ. The significant differences of gene relative abundance between feedstock and digestate samples were determined by the Wilcoxon signed-rank test at *P* < 0.05 [*P*-values were adjusted by Benjamini-Hochberg (BH) method]. The relationships between BGP operating temperature and the difference of ARG or pathogen relative abundance in digestate minus that in feedstock were analyzed by Spearman correlations using the R package PYSCH.

## Results

### A Comprehensive Gene Catalog of Feedstock Metagenome

**FIGURE 1:**
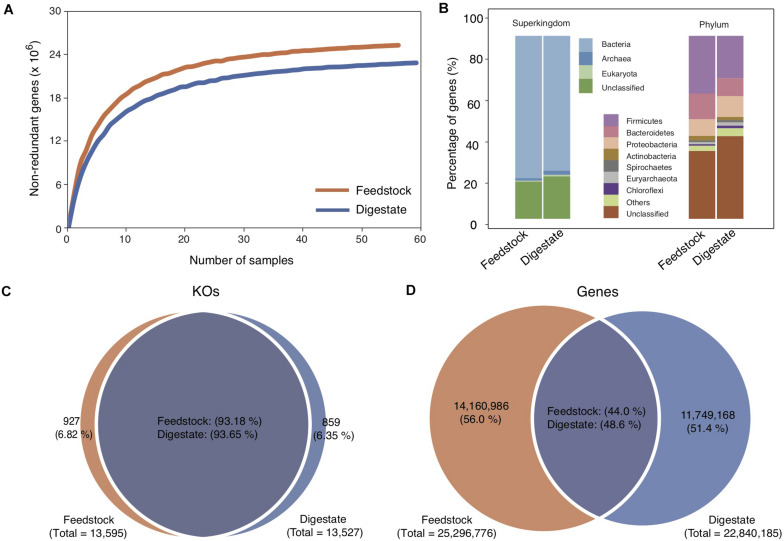
Comparison of microbial gene catalogs of feedstock and AD digestate. **(A)** Rarefaction curve based on the gene profile of all samples. The two curves approach saturation at the end of sampling. **(B)** Taxonomic annotation of the gene catalogs at the superkingdom and phylum levels. **(C,D)** Venn diagrams of shared KOs and genes between the two gene catalogs. The gene catalog of AD digestate was cited from a previous study (Ma et al., 2021).

Feedstock samples were collected before they were pumped into anaerobic digesters (Supplementary Figure 1), and a total of 56 feedstock samples were collected from the 56 full-scale biogas plants (Ma et al., 2021), with one sample from each plant. Deep sequencing of the 56 metagenomes generated a total of 1,716 Gb of high-quality data, with an average of 31 Gb per sample. Based on the assembled contigs with an average N50 length of 3,358 bp, we got a non-redundant Microbial Gene Catalog of Feedstock (MGC-F) containing a total of 25,296,776 genes, with an average open reading frame length of 770 bp and a full-length gene percentage of 53.44% (Table 1), which were comparable to those of Microbial Gene Catalog of AD (MGC-A) (Ma et al., 2021). A rarefaction analysis including all samples revealed a curve approached saturation with the increase of sample number (Figure 1A), suggesting that MGC-F covered the vast majority of microbial genes in these samples.

**TABLE 1:** Comparison of microbial gene catalog of feedstock (MGC-F) and AD digestate (MGC-A).

**Statistics**	**MGC-F**	**MGC-A**^‡^
Number of samples	56	59
Sequencing data (Gb)	1,716	1,817
Average contig N50 (bp)	3,358	3,986
Number of non-redundant genes	25,296,776	22,840,185
Average ORF length (bp)	770	790
Percentage of complete genes (%)	53.44	56.45

*^‡^The statistics for the gene catalog of AD digestate were downloaded from a previous study (Ma et al., 2021).*

Taxonomic annotation of MGC-F showed that 79.63% of the genes were classified at the superkingdom level, a little higher than that for MGC-A (76.73%) (Ma et al., 2021). Of these, more than 97% were assigned to *Bacteria*, and the remaining genes were assigned to *Archaea* (1.65%) and *Eukaryote* (0.78%). Similar to MGC-A, the predominant bacterial phyla were *Firmicutes* (31.52%), *Bacteroidetes* (13.89%), and *Proteobacteria* (9.18%) (Figure 1B), though higher proportion of genes were assigned to *Firmicutes* in MGC-F (Ma et al., 2021). At lower taxonomic levels, there were only 14.87 and 1.05% of the genes were assigned to specific genera and species, respectively.

Functional classification of MGC-F showed that 56.93% of the genes were annotated with KEGG orthologous groups (13,595 KOs), which are comparable to those of MGC-A (Ma et al., 2021). By comparing the pairwise overlap of KOs between the two gene catalogs, we found that ∼ 93% of KOs were shared between MGC-F (93.2%) and MGC-A (93.7%) (Figure 1C), which were higher than that for genes ∼ 46% shared between MGC-F (48.6%) and MGC-A (44.0%) (Figure 1D). Among these shared KOs, about 43.7% of KOs were unknown, while about 6.5, 4.9, 4.8, 4.6, and 3.0% of known KOs were assigned to top five of the KEGG pathways of signal transduction, amino acid metabolism, membrane transport, carbohydrate metabolism, metabolism of cofactors, and vitamins, respectively. However, for 927 unique KOs in feedstock samples, they were assigned to about 880 unique orthologous groups.

### Microbes and Functional Pathways for Methane Production Enriched From Feedstock to AD Digestate

**FIGURE 2:**
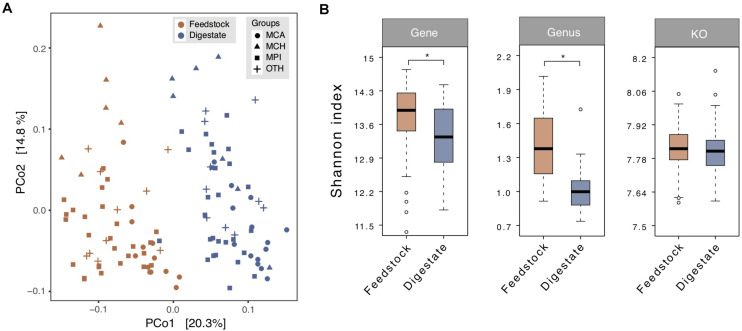
Comparison of microbial diversity between the microbial gene catalogs of feedstock and AD digestate. **(A)** Principal coordinate analysis (PCoA) at the genus level based on Bray-Curtis dissimilarity. The plots were separated into two clusters according to the feedstock and digestate. MCA, cattle manure biogas plants (BGPs); MCH, chicken manure BGPs; MPI, pig manure BGPs; OTH, BGPs with other feedstocks. **(B)** Microbial diversity (Shannon-Wiener index) at gene, genus, and KO levels. Asterisks denote significant difference between feedstock and digestate (adjusted *P* < 0.05). The relative gene abundance data for the gene catalog of AD digestate was downloaded from a previous study (Ma et al., 2021).

In general, feedstock for anaerobic digestion, consisting of a complex mixture of microorganisms, organic matters, and inorganic materials, is an essential factor that drives microbial community variation in anaerobic digesters (Zhang et al., 2014). Principal coordinate analysis (PCoA) based on Bray-Curtis dissimilarity at genus level showed a clear separation between the samples derived from feedstock and AD digestate (Figure 2A), displaying the obviously different microbial communities. Alpha-diversity analysis (Shannon-Wiener index) revealed that the microbial diversity of feedstock samples was higher than that of AD digestate samples at the gene (adjusted *P* < 0.05), genus (Wilcox rank sum test *P* < 0.05), and KO (adjusted *P* = 0.12) levels (Figure 2B).

**FIGURE 3:**
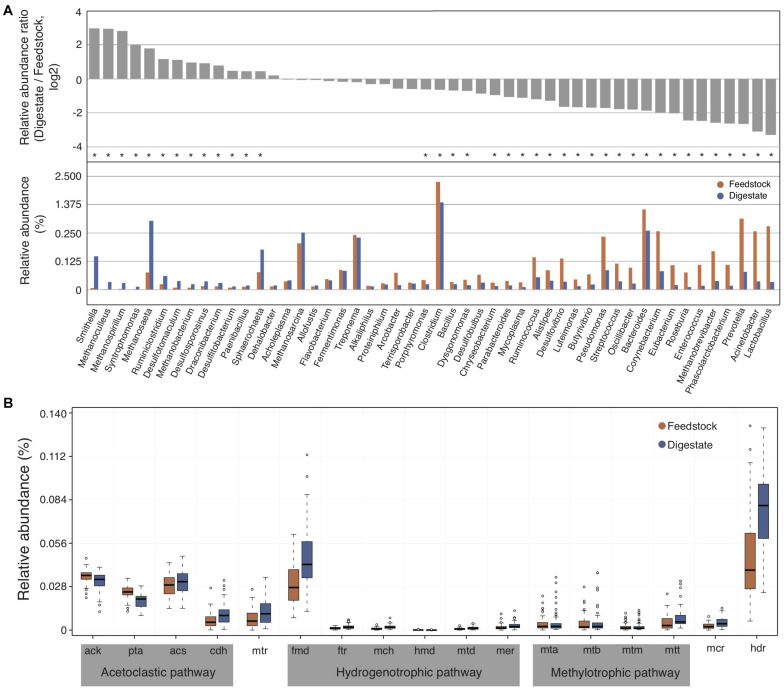
The changes of microbial communities from feedstock to AD digestate. **(A)** The relative abundance ratio of each selected genus in digestate to that in feedstock (upper part) and the relative abundance of these genera (lower part). The median value was showing. Asterisks denote significant difference between feedstock and digestate (adjusted *P* < 0.05). **(B)** Relative abundance of genes involved in the three methanogenesis pathways of acetoclastic, hydrogenotrophic, and methylotrophic methanogenesis. Genes involved in only one pathway were marked by gray background, while genes shared by two (*mtr*) or all three pathways (*mcr* and *hdr*) were not marked. The relative gene abundance data for the gene catalog of AD digestate was downloaded from a previous study (Ma et al., 2021).

To compare the differences of microbial communities between the feedstock and digestate of AD, we used the relatively abundant top 50 genera in digestate samples, which accounted for about 90% of the relative abundance of annotated genera. In brief, the relative abundance ratio of each genus in digestate to that in feedstock (digestate/feedstock) for each biogas plant was calculated, and then the median ratio value of all these 56 biogas plants was counted. As a result, the relative abundance of only 14 genera in AD digestate samples were higher than that of feedstock samples (with median ratio value > 1; Figure 3A and Supplementary Figure 2), indicating that only a small number of genera were enriched in AD. These enriched genera include several methanogens like *Methanoculleus*, *Methanospirillum*, *Methanosaeta*, and *Methanobacterium* (Figure 3A), and their relative abundance in AD digestates were significantly (adjusted *P* < 0.05) higher than that of feedstock samples, suggesting their importance to the AD process. In addition, two syntrophic bacteria of *Smithella* and *Syntrophomonas* were also significantly (adjusted *P* < 0.05) enriched (Figure 3A). They were reported to syntrophically oxidize propionate and butyrate in association with methanogens, respectively (Xia et al., 2019).

It is commonly accepted that methanogenic microorganisms are essential for AD process, and methanogenesis can occur via three main pathways, namely acetoclastic, hydrogenotrophic, and methylotrophic methanogenesis (Wintsche et al., 2018). The three methanogenesis pathways share some genes such as methyl-CoM reductase (*mcr*) and H4MPT-methyltransferase (*mtr*), but most of genes were unique in each methanogenesis pathway (Supplementary Figure 3). The relative abundance of most genes involved in acetoclastic methanogenesis pathway were higher than that in hydrogenotrophic and methylotrophic methanogenesis pathways in both feedstock and AD digestate (Figure 3B). In addition, the relative abundance of most genes involved in methanogenesis increased from feedstock to AD digestate, consistent with that many methanogens were increased from feedstock to AD digestate (Figure 3A).

To compare the microbial communities between the feedstock and AD digestate at genome level, metagenome-assembled genomes (MAGs) were constructed for feedstock samples. As a result, a total of 11,198 MAGs were generated from all 56 samples, with 3,015 MAGs have Completeness ≥80% and contamination ≤10%. After filtering the redundant genomes of the same species, a final of 2,101 representative MAGs were obtained, including 1,001 (47.6%) MAGs with Completeness ≥90% and contamination ≤5%. Taxonomic annotation of the MAGs revealed that 97.3 and 2.7% of MAGs were assigned to Bacteria and Archaea, respectively, which were consistent to that of AD digestate samples (Ma et al., 2021). The predominant phyla were *Firmicutes* (45.7%), *Bacteroidetes* (24.8%), and *Proteobacteria* (5.5%), which were consistent with the result derived from the gene catalog. In addition, MAGs generated from AD digestate samples (Ma et al., 2021) were compared to those from feedstock samples, and about 23% of MAGs in AD digestate were identified as the same species to those in feedstock samples (with ANI value > 95% and genome coverage >50% between the two MAGs). These results were consistent with fact that a huge number of AD populations were migrated from upstream feedstocks in BGPs (Mei et al., 2017).

### Anaerobic Digestion Reduces the Dissemination of ARGs From Manure Wastes

**FIGURE 4:**
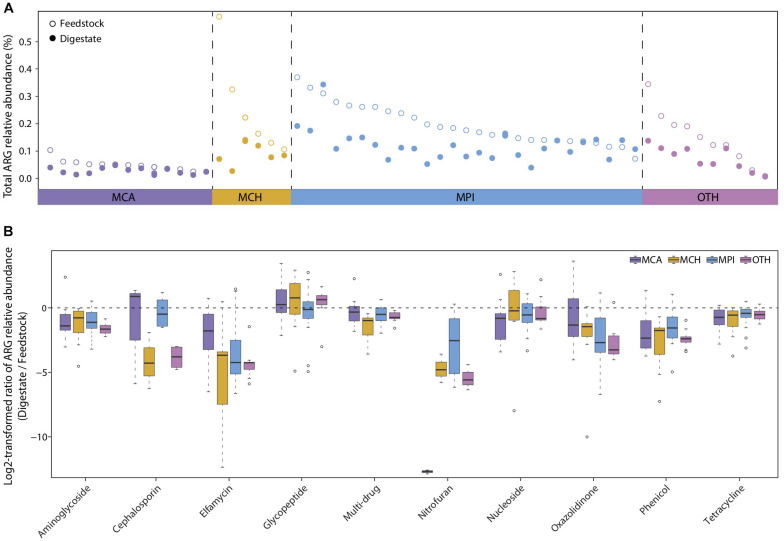
Reduction of antibiotic resistance genes (ARGs) from feedstock to AD digestate. **(A)** Total relative abundance of ARGs in digestate and the corresponding feedstock of each BGP. MCA, cattle manure BGPs; MCH, chicken manure BGPs; MPI, pig manure BGPs; OTH, BGPs with other feedstocks. Hollow circles represent feedstock samples, and solid circles represent digestate samples. **(B)** Relative abundance ratio of 10 major ARG types in digestate to that in the corresponding feedstock of each BGP. Asterisks denote significant difference between feedstock and digestate (adjusted *P* < 0.05). The relative gene abundance data for the gene catalog of AD digestate was downloaded from a previous study (Ma et al., 2021).

The dissemination of antibiotic resistance genes (ARGs) from manure wastes to natural environment has become a global health issue (Youngquist et al., 2016; Wu et al., 2018). Existing studies have proposed AD as a promising method to reduce ARGs in manure (Wallace et al., 2018; Wu et al., 2018). However, only a few researches have studied the occurrences and changes of diverse ARGs in several full-scale BGPs treating manure (Luo et al., 2017), and not all ARGs can be effectively reduced in AD process. Thus, we used the metagenomic data to investigate the prevalence of all known ARGs in both AD digestates and the corresponding feedstocks of 56 BGPs. According to their substrate types, the 56 BGPs were divided into four groups: MCA (13 cattle manure BGPs), MCH (6 chicken manure BGPs), MPI (27 pig manure BGPs), and OTH (10 BGPs with other substrates) (Supplementary Table 1). Overall, AD reduced approximately 40% of the ARGs present in different feedstocks, with medians of the total ARGs relative abundance decreased by 51, 62, 36, and 48% in MCA, MCH, MPI, and OTH, respectively Figure 4A, possibly due to the decreased selective pressure on ARGs caused by the degradation of antibiotics in AD (Cheng et al., 2016; Turker et al., 2018; Huang et al., 2019). Besides, the changes of feedstock dilution, temperature, pH, ammonia concentration, may also influence the prevalence of ARGs. In particular, the decrease of most ARGs by AD was more evident in BGPs operated at relatively high temperatures typically 30–40°C (Supplementary Figure 4 and Supplementary Table 2).

AD significantly reduced (*P* < 0.05) the relative abundances of 15, 21, 23, and 20 ARG types in, respectively MCA, MCH, MPI, and OTH (Supplementary Table 3), and the reduction rates varied greatly among different ARG types. The highly abundant (>0.001%) ARGs dominated in all BGPs were the widely reported tetracycline, multi-drug, aminoglycoside, lincosamide, and macrolide resistance genes (Luo et al., 2017; Huang et al., 2019), with reduction rates of 20–70% in AD (Figure 4B and Supplementary Figure 5). Similarly, the reduction of some genes conferring tetracycline, aminoglycoside, and macrolide resistance was also observed in AD reactors treating cattle manure, domestic wastewater, and pig manure (Resende et al., 2014; Christgen et al., 2015; Ben et al., 2017). The less abundant (< 0.0001%) ARGs were cephalosporin, elfamycin, nitrofuran, nitroimidazole, and streptogramin resistance genes, with reduction rates higher than 95% in AD (Figure 4B and Supplementary Table 3). Moreover, we have detected the rarely reported phenicol and oxazolidinone resistance genes and they were also reduced by about 70% in AD. However, nucleoside and glycopeptide resistance genes were not reduced in AD (Figure 4B and Supplementary Table 3), similar to the persistence of sulfonamide and chloramphenicol ARGs in AD (Christgen et al., 2015). In summary, our results show that most ARGs, in particular the less abundant ARGs, are effectively reduced by AD, and the persistence of nucleoside and glycopeptide resistance genes deserves attention in future researches.

### Elimination of Common Pathogens in Manure by Anaerobic Digestion

**FIGURE 5:**
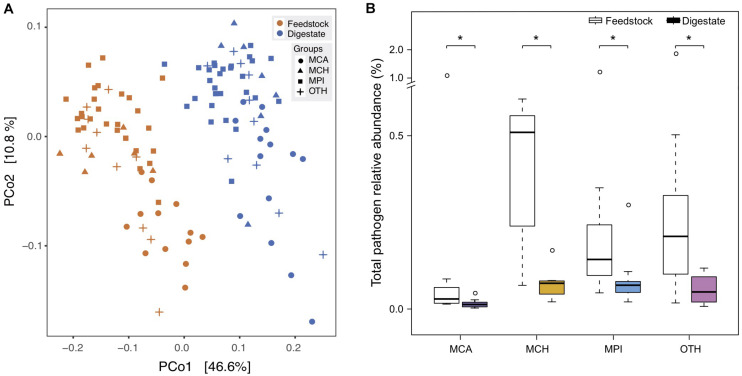
Reduction of pathogenic bacteria from feedstock to AD digestate. **(A)** Principal coordinate analysis (PCoA) based on Bray-Curtis dissimilarities of pathogens. MCA, cattle manure BGPs; MCH, chicken manure BGPs; MPI, pig manure BGPs; OTH, BGPs with other feedstocks. Hollow circles represent feedstock samples, and solid circles represent digestate samples. **(B)** Total relative abundance of 79 pathogens in the feedstock and digestate. Asterisks denote significant difference between feedstock and digestate (adjusted *P* < 0.05). The relative gene abundance data for the gene catalog of AD digestate was downloaded from a previous study (Ma et al., 2021).

The outbreak of food-borne diseases threatens human health worldwide, partly due to the spreading of human pathogenic bacteria from animal manure (Altmann et al., 2011; Authority et al., 2017). Manure treatment by AD has been reported to reduce some culturable human pathogens (Massé et al., 2011; Fröschle et al., 2015; Orzi et al., 2015), while some other pathogens still persist in the digestate of AD (Owamah et al., 2014). Up to now, there lacks a comprehensive assessment on the risks of various human pathogens in AD, so we used the metagenomic data to investigate the change of all pathogens in the AD process of full-scale biogas plants. In total, we identified 79 human pathogenic bacteria (species) in the 56 BGPs, and digestates and corresponding feedstocks showed distinct difference in pathogen composition (Figure 5A). The medians of total pathogen relative abundance were reduced by 55, 85, 52, and 76% in MCA, MCH, MPI, and OTH, respectively (Figure 5B). Particularly, the BGP operating temperature showed negative correlations with the reduction of most pathogens in AD (Supplementary Figure 6 and Supplementary Table 4). On average, AD removed about 55% of the detected pathogens in different feedstocks, possibly because most pathogens are aerobic or facultative aerobic bacteria (Tang et al., 2015) whose growth is restricted by anaerobic environments and unfavorable high temperatures like 30–40°C. In addition, the abundance of pathogens may also be affected by the changes of feedstock dilution, pH, ammonia concentration, etc.

For most of the highly abundant (0.001% ∼ 0.01%) pathogens in AD, their relative abundances were significantly (*P* < 0.05) reduced by 30–99% during AD process, and these pathogens included the widely reported *Acinetobacter baumannii*, *A. lwoffii*, *Campylobacter coli*, *Clostridium perfringens*, *Enterococcus faecalis*, *E. faecium*, *Escherichia coli*, *Listeria monocytogenes*, and *Salmonella enterica* (Eikmeyer et al., 2013; Fröschle et al., 2015), and the rarely reported *Klebsiella pneumoniae*, *Pseudomonas aeruginosa*, *Staphylococcus aureus*, and *Streptococcus anginosus* (Supplementary Table 5 and Supplementary Figure 7). Previously, the reduction of culturable species of *Clostridium*, *Acinetobacter*, *Escherichia*, *Enterococcus*, and *Salmonella* was also observed in AD reactors treating cattle manure (Resende et al., 2014; Qi et al., 2019). However, for other highly abundant pathogens of *Bacteroides fragilis*, *Fusobacterium necrophorum*, and *Streptococcus pneumoniae*, their relative abundances were not reduced during AD process (Supplementary Table 5 and Supplementary Figure 7). Therefore, the potential spreading of anaerobic *B. fragilis*, *F. necrophorum* and facultative anaerobic *S. pneumoniae* from manure or digestate deserves serious attention, for that they are the leading causes of intraabdominal infections, pharyngotonsillitis, pneumonia, and bacterial meningitis in children and the elderly (Sears et al., 2006; Janoff and Musher, 2015; Holm et al., 2016).

## Discussion

Given the importance of anaerobic digestion (AD) in resource recycling and energy recovery, we constructed the first comprehensive microbial gene catalog of feedstock, and made a comprehensive comparison between the metagenome of feedstock and AD digestate. Our result showed that the predominant phyla in feedstock were *Firmicutes*, *Bacteroidetes*, and *Proteobacteria*, which were similar to that of AD digestate, and the microbial diversity of feedstock samples was higher than that of AD digestate samples. In addition, the relative abundance of most genes involved in methanogenesis increased from feedstock to AD digestate, consistent with that many methanogens were increased from feedstock to AD digestate. Besides, the obvious reduction of antibiotic resistance genes (ARGs) and pathogenic bacteria in AD was observed in almost all BGPs, suggesting the potential of AD to help control the environmental threat of ARGs and pathogens.

This study deepens the understanding of microbial compositions and functions in the feedstock and AD digestate, which has the potential to improve biogas production that can supply more clean energy, reduce the dependence on fossil fuels, and accelerate the development of the recycling economy. Furthermore, a good knowledge of the microbe-mediated process from organic matter to methane, a greenhouse gas (GHG) with a strong global warming effect, is crucial for the guidance of reducing methane emissions from various environments, such as livestock rumen, paddy field and peatland. Therefore, the results from this study will not only benefit the environment and socioeconomy but also provide some clues to resolving the challenge of global warming.

## Data Availability Statement

The datasets presented in this study can be found in online repositories. The names of the repository/repositories and accession number(s) can be found below: https://www.ncbi.nlm.nih.gov/genbank/, PRJNA681092.

## Author Contributions

FJ, YZ, SM, YH, HF, and QL collected the samples and performed the experiments. FJ, SW, YZ, and HW analyzed the data. AW and HL provide helpful suggestions. FJ, SW, and YZ wrote the raw manuscript. LC, YD, and WF conceived the study, designed the experiments, and revised the manuscript. All authors read and approved the final manuscript.

## Conflict of Interest

The authors declare that the research was conducted in the absence of any commercial or financial relationships that could be construed as a potential conflict of interest.

## Footnotes

1 https://github.com/arpcard/rgi

2 https://sourceforge.net/projects/bbmap/

## Abbreviations

AD: anaerobic digestion
ARG: antibiotic resistance gene
BGP: biogas plant
CARD: Comprehensive Antibiotic Resistance Database
GHG: greenhouse gas
Gb: gigabase
KO: KEGG orthologous group
MCA: cattle manure biogas plants
MCH: chicken manure biogas plants
MGC-A: Microbial Gene Catalog of anaerobic digestion
MGC-F: Microbial Gene Catalog of Feedstock
MPI: pig manure biogas plants
ORF: open reading frame
OTH: biogas plants with other feedstocks
PCoA: principal coordinate analysis.
