# Supplementary material for: Variation of Metagenome From Feedstock to Digestate in Full-Scale Biogas Plants
Source: Front Microbiol. 2021 May 28;12:660225. doi: 10.3389/fmicb.2021.660225 (PMC8193575; doi:10.3389/fmicb.2021.660225)
Supplement: Supplementary file 6 [file Data_Sheet_1.pdf]

Supplementary Material

1 Supplementary Figures

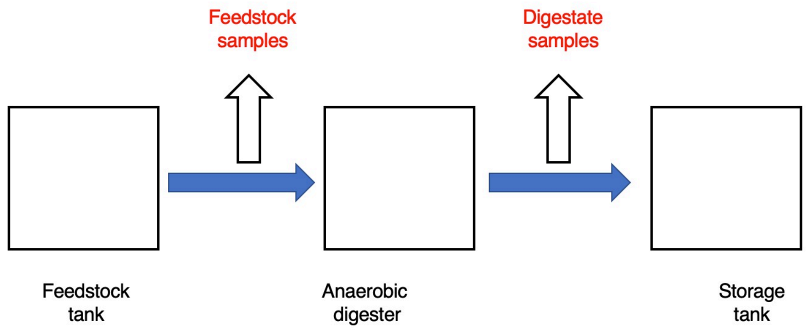

Figure S1. Biogas plants process and sampling points for feedstock and digestate samples.

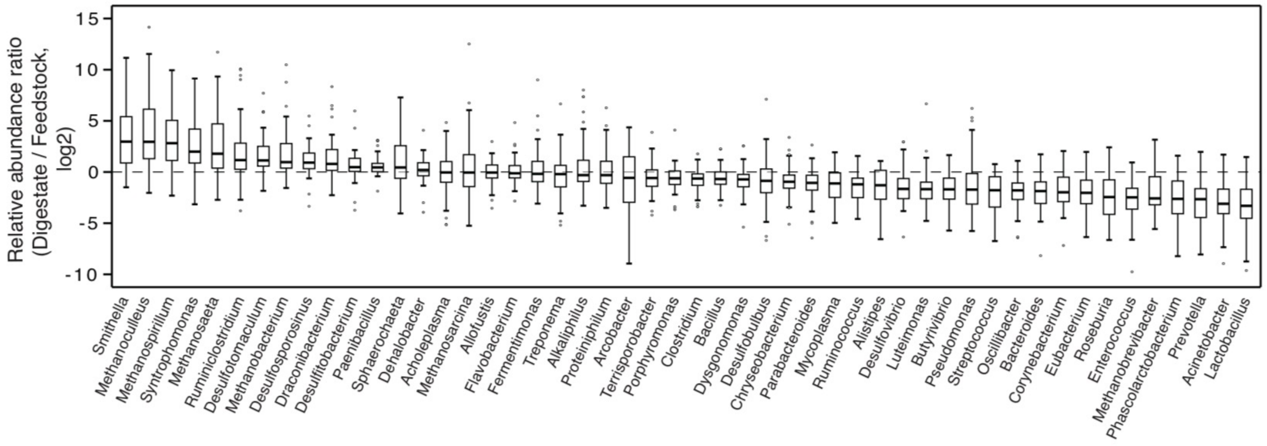

Figure S2. Boxplot showing the relative abundance ratio of each selected genus in digestate to that in feedstock. Box plots show medians ± interquartile ranges (IQR) and 1.5 IQR ranges (whiskers), with outliers denoted by open black circles.

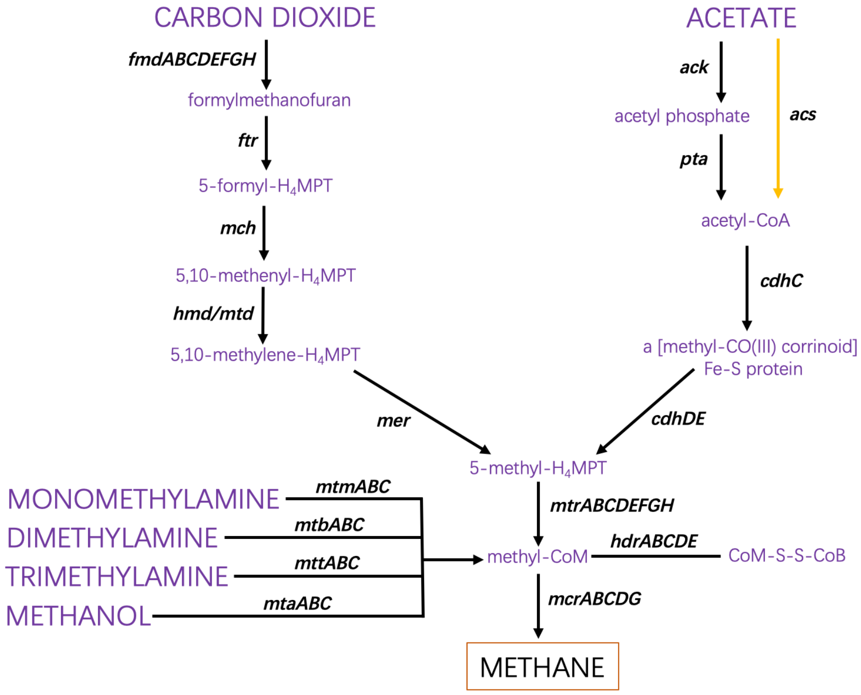

Figure S3. The three methanogenesis pathways, namely acetoclastic, hydrogenotrophic and methylotrophic methanogenesis. Yellow arrow represents another pathway from acetate to acetyl-CoA. ***ack***, acetate kinase; ***pta***, phosphate acetyltransferase; ***acs***, acetyl-CoA synthetase; ***cdhC***, CO dehydrogenase/acetyl-CoA synthase; ***cdhAB***, CO dehydrogenase/acetyl-CoA synthase; ***fmdABCDEFGH***, formylmethanofuran dehydrogenase; ***ftr***, formylmethanofuran-H_4MPT formyltransferase; ***mch***, methenyl-H_4MPT cyclohydrolase; ***hmd/mtd***, H2-forming N5, N10-methylene-H4MPT dehydrogenase/ methylene-5,6,7,8H4MPT dehydrogenase; ***cdhDE***, CO dehydrogenase/acetyl-CoA synthase; ***mtrABCDEFGH***, H_4MPT S-methyltransferase; ***hdrABCDE***, CoB-CoM heterodisulfide reductase; ***mer***, 5,10-methylene-H_4MPT reductase; ***mcrABCDG***, methyl-CoM reductase; ***mtmABC***, monomethylamine methyltransferase; ***mtaABC***, methanol-cobalamin methyltransferase; ***mtbABC***, dimethylamine methyltransferase; ***mttABC***, trimethylamine methyltransferase.

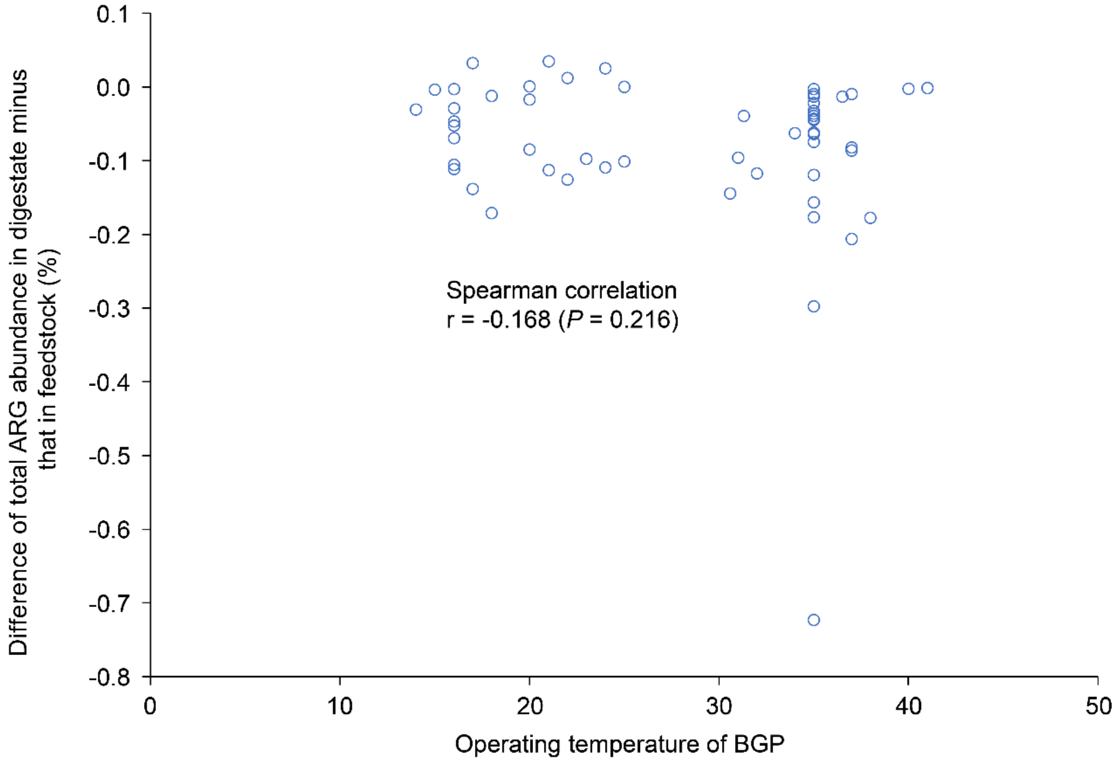

Figure S4. Relationship of the operating temperature with the difference of total relative abundance of antibiotic resistance genes (ARGs) in feedstock minus that in feedstock of 56 full-scale biogas plants (BGPs). Each circle refers to a BGP.

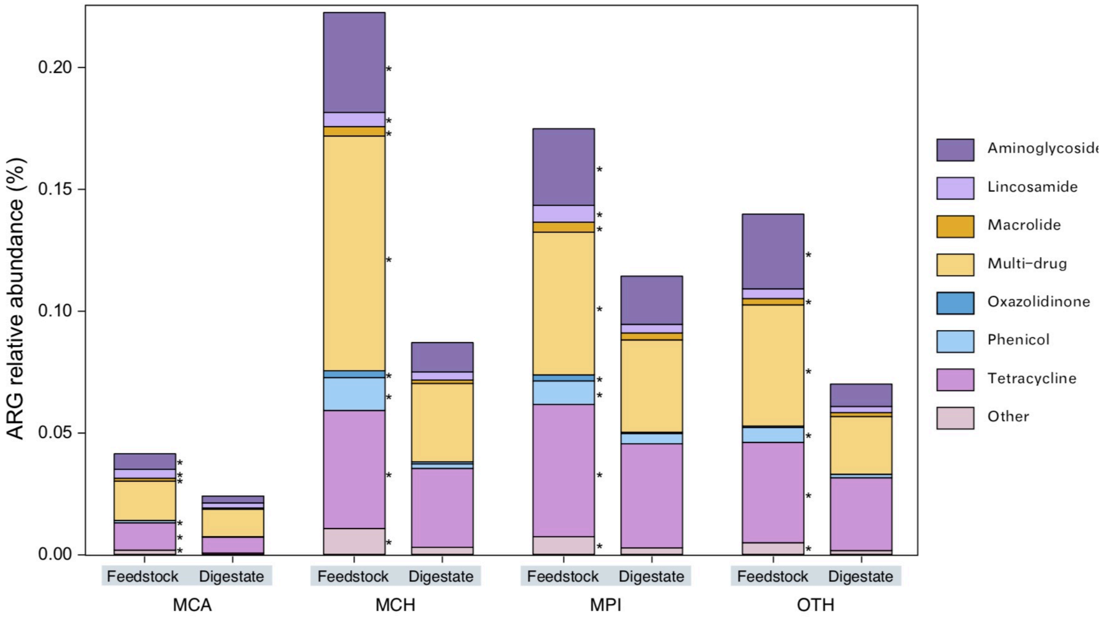

Figure S5. Comparison of antibiotic resistance gene (ARG) types between AD digestate and corresponding feedstock samples. Accumulated relative abundance medians of 7 major and other ARG types in the feedstock and digestate samples. Asterisks near stacked bars indicate significantly higher relative abundances of the corresponding ARGs in the feedstock than those in the digestate by Wilcoxon signed-rank test at $P < 0.05$. P-values were adjusted by Benjamini-Hochberg (BH) method. (PDF 167kb)

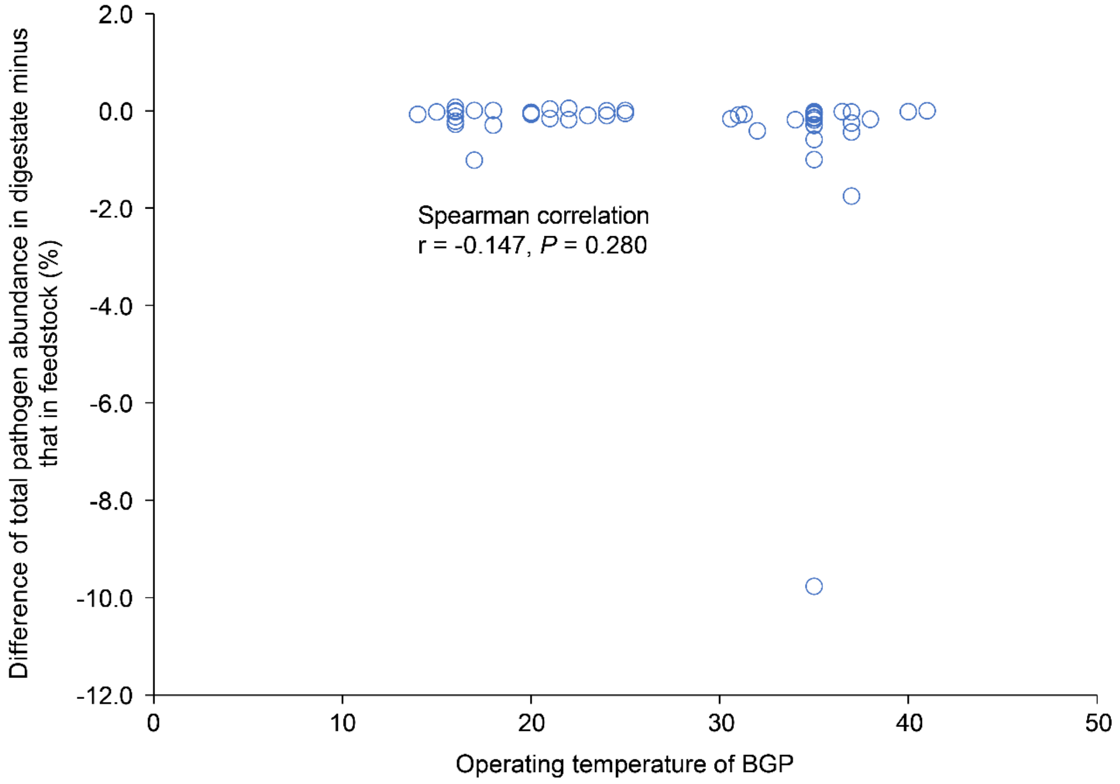

Figure S6. Relationship of the operating temperature with the difference of total relative abundance of human pathogens in feedstock minus that in feedstock of 56 full-scale biogas plants (BGPs). Each circle refers to a BGP.

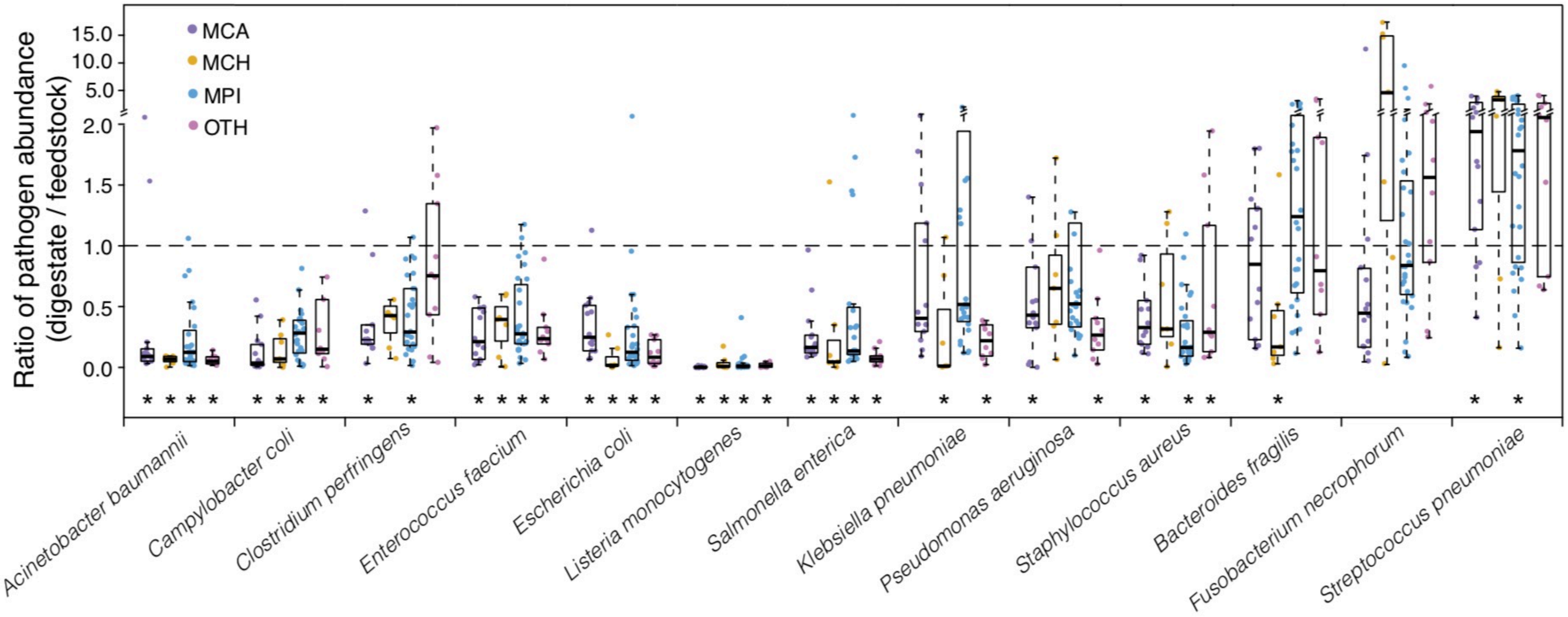

Figure S7. Relative abundance ratio of 13 major pathogens in digestate to the corresponding feedstock of each BGP. Box plots show medians ± interquartile ranges (IQR) and 1.5 IQR ranges (whiskers), with outliers denoted by open black circles. Asterisks denote significantly increased or reduced pathogens in AD (Wilcoxon signed-rank test at $P < 0.05$, P-values were adjusted by Benjamini-Hochberg (BH) method)

2 Supplementary Tables

Table S1. Background information of the sampled biogas plants.

Table S2. Spearman correlations between the operating temperature and the difference of the relative abundance of 29 types of antibiotic resistance genes (ARGs) in digestate minus that in feedstock of 56 full-scale biogas plants (BGPs).

Table S3. Wilcoxon signed-rank test of the relative abundance of 29 types of antibiotic resistance genes (ARGs) between feedstock and digestate samples of 56 full-scale biogas plants (BGPs). *P*-values were adjusted by Benjamini-Hochberg (BH) method.

Table S4. Spearman correlations between the operating temperature and the difference of the relative abundance of 79 pathogen species in digestate minus that in feedstock of 56 full-scale biogas plants (BGPs).

Table S5. Wilcoxon signed-rank test of the relative abundance of 79 pathogens between feedstock and digestate of 56 BGPs. *P*-values were adjusted by Benjamini-Hochberg (BH) method.
